# Estimating reference intervals from an IPD meta-analysis using quantile regression

**DOI:** 10.1186/s12874-024-02378-0

**Published:** 2024-10-26

**Authors:** Ziren Jiang, Haitao Chu, Zhen Wang, M. Hassan Murad, Lianne K. Siegel

**Affiliations:** 1 Division of Biostatistics and Health Data Science, University of Minnesota, 2221 University Ave. SE., Ste. 200, Minneapolis, MN 55414 USA; 2 Statistical Research and Data Science Center, Pfizer Inc., New York, USA; 3 Evidence-Based Practice Center, Robert D. and Patria E. Kern Center for the Science of Health Care Delivery, Mayo Clinic, Rochester, Minnesota USA

**Keywords:** Reference interval, Quantile regression, Meta-analysis, Individual participant data, Bootstrap

## Abstract

**Background:**

Reference intervals, which define an interval in which a specific proportion of measurements from a healthy population are expected to fall, are commonly used in medical practice. Synthesizing information from multiple studies through meta-analysis can provide a more precise and representative reference interval than one derived from a single study. However, the current approaches for estimating the reference interval from a meta-analysis mainly rely on aggregate data and require parametric distributional assumptions that cannot always be checked.

**Methods:**

With the availability of individual participant data (IPD), non-parametric methods can be used to estimate reference intervals without any distributional assumptions. Furthermore, patient-level covariates can be introduced to estimate personalized reference intervals that may be more applicable to specific patients. This paper introduces quantile regression as a method to estimate the reference interval from an IPD meta-analysis under the fixed effects model.

**Results:**

We compared several non-parametric bootstrap methods through simulation studies to account for within-study correlation. Under fixed effects model, we recommend keeping the studies fixed and only randomly sampling subjects with replacement within each study.

**Conclusion:**

We proposed to use the quantile regression in the IPD meta-analysis to estimate the reference interval. Based on the simulation results, we identify an optimal bootstrap strategy for estimating the uncertainty of the estimated reference interval. An example of liver stiffness measurements, a clinically important diagnostic test without explicitly established reference range in children, is provided to demonstrate the use of quantile regression in estimating both overall and subject-specific reference intervals.

**Supplementary Information:**

The online version contains supplementary material available at 10.1186/s12874-024-02378-0.

## Introduction

A reference interval, defined as “the values between which the test results of a specified percentage (usually 95%) of apparently healthy individuals would fall” [1, 2], plays an important role in medical practice [3]. Estimating a reference interval from a single study may be limited by a relatively small sample size or not generalize to a broader population [4]. Therefore, synthesizing the information from multiple studies in a meta-analysis can provide a more precise and representative reference interval.

Siegel et al., [5] recently pointed out some common misunderstandings in the estimation of reference intervals from meta-analyses and proposed three different methods using aggregate data (mean, standard deviation, and sample size of each study) to estimate the reference interval from a random effects meta-analysis. Cao et al. [4] further proposed two methods (including the empirical method from Siegel et al.) extending the fixed effects meta-analysis model using the aggregate data. These methods assume the data within each study follow some parametric distribution (normal, log-normal, or other two parameter distributions). However, this distributional assumption often cannot be checked with only the aggregate data. Individual participant data (IPD) allow the assessment of the within-study distributional assumption; the previously proposed methods for estimating the reference interval can then be used after aggregating IPD. Siegel et al. [3] also demonstrated alternative versions of these methods fit directly using the IPD. Alternatively, Khoshdel et al. [6] used fractional polynomial functions to estimate the age-specific reference interval for pulse wave velocity through a meta-analysis.

Quantile regression [7] has been widely used in economics, statistics, and medical research [8]. It estimates the conditional quantile instead of the usual conditional mean as in ordinary linear regression [9] and does not require any distributional assumptions. Thus, it can be more flexible and robust for some non-normal scenarios. See the Supplementary Material for further details. Quantile regression has been applied to estimate the reference interval for a single data set [8, 10]. Additionally, it has been used to estimate growth charts [11], normal response amplitudes of nerves conditions [12], and a reference interval for the Singapore Caregiver Quality of Life Scale (SCQOLS) [13]. We introduce quantile regression for estimating the reference interval from an IPD meta-analysis as a complement to the other parametric methods, especially when avoiding a within-study distributional assumption is desired. We also demonstrate how to account for patient-level differences and estimate personalized reference intervals using covariates such as age or sex.

## Methodology

### Meta-analysis models

Assume there are $$\:K$$ studies in the meta-analysis, where each study contains $$\:{n}_{i},$$$$\:i=1,\dots\:,\:K,$$ subjects. Denote the underlying mean of study $$\:i$$ as $$\:{\theta\:}_{i}$$. Traditionally, there are three meta-analysis models to explain the difference of the study means. The common effect model assumes a common true mean for each study and attributes any differences to sampling variation. The random effects model assumes that the true means of each study differ and follow a common (usually normal) distribution. The overall population is then defined hierarchically through the distribution of the study means and the subsequent distribution of the measurements within each study [14, 15]. The number of studies in the meta-analysis is commonly relatively small (around 5 or less); assuming a parametric distribution for the study means in this setting may be undesirable, as this assumption is hard to defend [16] and the between study variance is difficult to estimate. In these cases, the fixed effects model is often preferable; this also assumes that each study mean is different, though unlike the random effects model, it does not have a distributional assumption on the study means [17–19]. The fixed effects model instead assumes the collection of studies is representative of the overall population [20]. The overall population is thus defined as the aggregated population of the included studies. Then, the cumulative distribution function (CDF) of the overall population $$\:F$$ can be estimated as the mixture of each study’s distribution:

$$\:\widehat{F}={\sum\:}_{i=1}^{K}\frac{{w}_{i}{\widehat{F}}_{i}}{{\sum\:}_{i=1}^{n}{w}_{i}}$$,

where $\:{w}_{i}$ is the weight for study $\:i$. Some common choices for the weights include sample size weights or the inverse variance weights. Denote the true overall population as $\:F$; the $\:95\%$ reference interval can then be defined as $\:[L,\:U]$ where $\:L\:$and $\:U$ are the 0.025 and 0.975 quantiles of $\:F$ respectively.

Note that, if we use study $\:i$’s empirical CDF $\:{\widehat{F}}_{i}$ to estimate its distribution function $\:{F}_{i}$, the estimated overall distribution $\:\widehat{F}={\sum\:}_{i=1}^{K}\frac{{w}_{i}{\widehat{F}}_{i}}{{\sum\:}_{i=1}^{n}{w}_{i}}$ with $\:{w}_{i}={n}_{i}$ as the sample size weights is the same as the empirical distribution of the pooled data. Therefore, since the quantile regression provides a consistent estimator of the conditional $\:\tau\:=0.025$ and $\:\tau\:=0.975$ quantiles for the pooled sample [21, 22], it is also a consistent estimator of the upper and lower bound of the $\:95\%$ reference interval defined previously.

### Using quantile regression to estimate the reference interval

We use sample size weights for each study ($\:{w}_{i}={n}_{i})$ and let $\:{\widehat{F}}_{i}$ be the empirical distribution of study $\:i$’s sample. Once we have the individual participant data (IPD), the empirical distribution of the pooled IPD sample is the same as the estimated overall distribution $\:\widehat{F}={\sum\:}_{i=1}^{K}{w}_{i}{\widehat{F}}_{i}/{\sum\:}_{i=1}^{K}{w}_{i}$. Therefore, performing quantile regression on the pooled IPD sample is similar to using a fixed effects model with respect to the study means. The upper and lower bounds of the $\:95\%$ reference interval can be estimated by setting $\:\tau\:=0.025$ and $\:\tau\:=0.975$ in two quantile regression models, respectively. Covariates, such as age, can be applied to investigate their relationship with the estimated reference interval. Instead of specifying the mean and variance of the measurement’s distribution (and estimating how covariates influence them), quantile regression directly estimates the quantiles of the target measurement’s distribution and thus more flexibly describes how these covariates relate to the reference interval. The reference interval derived from the pooled IPD thus reflects the normative range of the measurement across the target population, which is defined as the combined populations of each included study under the fixed effects model. If no covariates are used in the quantile regression, the estimated limits of the $\:95\%$ reference interval are equivalent to the $\:\tau\:=0.025$ and $\:\tau\:=0.975\:$sample quantiles.

### Inference about the estimated reference interval

We use non-parametric bootstrap resampling methods to estimate the uncertainty in the limits of the estimated reference interval. Since the IPD are clustered by study, simply randomly sampling the pooled data with replacement does not consider the correlation and data structure within each study. The bootstrap method should account for this hierarchical structure of the IPD data. Both parametric and non-parametric bootstrap methods for clustered data have been proposed and studied in recent years [23–28]. However, unlike the common longitudinal data situation of a relatively large number of clusters and small number of observations within each cluster, meta-analysis typically contains fewer studies (i.e., clusters) and a relatively large number of observations (i.e., participants) within each study. Therefore, we use simulation studies to examine the performance of different bootstrap strategies.

For the non-parametric bootstrap, generally there are four potential strategies for bootstrapping the clustered data. The first layer of the data represents each study, and the second layer represents individuals within each study. Strategy 1 fixed the first layer (each study) without sampling (so that all studies are included) and for each study randomly samples the second layer (individual data within each study) with replacement, therefore we name it as the NY method, where N refers to “without sampling” and Y refers to “sampling with replacement”. Strategies 2 and 3 both sample the first layer with replacement; for each included study, strategy 2 then samples the second layer with replacement (named as the YY method) and strategy 3 without replacement (named as the YN method). Note that, for each included study, the resample of the second layer has the same sample size as the original study. Therefore, the sample without replacement just includes all subjects in the selected study. Strategy 4 ignores the structure of clustered data and simply samples the pooled dataset with replacement; therefore, we name it the “naïve method”.

For hierarchical data analyzed with random effects model, Ren et al. [28] advocate the use the YN strategy, claiming it mimics the variation properties of the data best. Alternatively, Field et al., [24] studied the asymptotic properties of different bootstrap methods and concluded that NY method is asymptotically consistent under an asymptotically infinite number of clusters under the random effects model. However, as mentioned earlier, in practice, the number of clusters in a meta-analysis is often small, and thus asymptotic results may not apply. In addition to non-parametric bootstrap methods, we also consider the cluster-robust bootstrap method proposed by Andreas Hagemann [29] for the quantile which is implemented in the R package “quantreg”. This wild bootstrap method does not directly resample from the observed data; it instead draws independent and identically distributed (iid) values with mean 0 and variance 1 and multiplies them by the residuals of the model to generate observations. They then use the generated observations to refit the model and produce the bootstrap sample. We refer to this as the “Wild” bootstrap strategy.

## Simulation

### Simulation methods

The setting of meta-analysis can involve extreme scenarios that may influence the relative performances of the different bootstrap methods when estimating standard errors and confidence intervals. For example, the number of studies is often very small (less than 10) with unbalanced sample sizes. Also, the quantiles to be estimated for a reference interval are typically very close to the tails and thus may be highly variable due to the relatively small sample size. Therefore, we conducted simulation studies to explore the performance of different bootstrap strategies in estimating the uncertainty of the quantile regression estimators for the reference interval limits.

We first simulated each study mean $\:{\theta\:}_{i},\:i=1,\dots\:,K$ according to a normal distribution, *θ*_i_∼ *N* (0, *τ*^2^), where $\:{\tau\:}^{2}=1$ represents the between study heterogeneity. Then, for each simulation replication, we fixed $\:{\theta\:}_{i}$ and generated $\:{Y}_{ij},\:i=1,\dots\:,K,\:j=1,\dots\:,{n}_{i}$, the individual observations from each study, according to a normal distribution with mean $\:{\theta\:}_{i}$ and standard deviation $\:{\sigma\:}^{2}=3.$ Here, the data generating mechanism follows the fixed effects model where we do not regenerate the underlying study means over the simulation replications. We also considered cases where observations followed a gamma distribution with shape parameter $\:\alpha\:={\theta\:}_{i}^{2}/{\sigma\:}^{2}$ and scale parameter $\:\beta\:={\sigma\:}^{2}/{\theta\:}_{i}$. We considered $\:K=5,\:10$ to represent situations with a limited number of studies. We also included both balanced and unbalanced cases for the number of participants in each study. For the balanced study cases, each$\:\:\:{n}_{i}=60\times\:m$ where $\:m=1,\:2,\:3$ be the multiplier of the sample size within each study; for the unbalanced cases, the sample sizes $\:{n}_{i}\:i=1,\dots\:,\:10$ were chosen as $\:\left(\text{20,20,40,40,60,60,80,80,100,100}\right)\times\:m$ if $\:K=10$ and $\:\left(\text{20,40,60,80,100}\right)\times\:m$ if $\:K=5$.

### Results

The simulation results can be seen in Tables 1, 2 and 3. For each bootstrap method, we calculated the averages of the estimated bootstrap standard errors of the upper reference interval limits (0.975 quantile) across the 1,000 replications. In Table 1, each result is compared with the empirical standard deviation of the estimated upper limits over the 1,000 replications. Additionally, 95% confidence intervals were calculated from the sample 0.025 and 0.975 quantiles of the bootstrap sample. The corresponding coverage rates are displayed in Table 2 to show the performance of each bootstrap confidence interval in maintaining the nominal coverage probability.

**Table 1:** Simulation results for the estimated standard error for the estimator of the 0.975 quantile. “NY”, “YY”, “YN”, “Wild” and “Naïve” are the methods described in Sect. 2.3. The “Emp” SE is the standard deviation of the simulated quantiles. Column “m” represents the multiplier of the number of subjects within each study ($\:60\times\:m$ subjects each study for the balanced case, for unbalanced case $\:\left(\text{20,20,40,40,60,60,80,80,100,100}\right)\times\:m$ if $\:K=10$ and $\:\left(\text{20,40,60,80,100}\right)\times\:m$ if $\:K=5$). “Balance” represents whether the sample sizes for each study is balanced or not. “Dist” represents the distribution within each study where “G” represent Gamma distribution and “N” represent normal distribution

*m*	Study #	Balance	Dist	Emp	NY	YY	YN	Wild	Naïve
1	5	Balanced	G	0.214	0.224	2.756	2.748	111.029	0.237
			N	0.174	0.174	0.400	0.361	13.544	0.177
		Unbalanced	G	0.196	0.202	2.978	2.971	160.658	0.208
			N	0.178	0.180	0.473	0.436	31.631	0.184
	10	Balanced	G	0.175	0.185	1.855	1.846	135.163	0.212
			N	0.126	0.129	0.364	0.341	13.871	0.133
		Unbalanced	G	0.182	0.184	1.897	1.892	160.124	0.211
			N	0.132	0.136	0.464	0.445	34.339	0.142
2	5	Balanced	G	0.154	0.159	2.750	2.747	113.935	0.168
			N	0.121	0.119	0.366	0.346	9.123	0.121
		Unbalanced	G	0.140	0.142	2.977	2.973	163.480	0.145
			N	0.120	0.125	0.439	0.418	32.783	0.128
	10	Balanced	G	0.126	0.131	1.848	1.848	139.972	0.149
			N	0.094	0.091	0.342	0.332	11.993	0.094
		Unbalanced	G	0.130	0.131	1.885	1.884	162.344	0.149
			N	0.096	0.095	0.443	0.433	35.185	0.099
4	5	Balanced	G	0.125	0.129	2.745	2.745	114.458	0.137
			N	0.100	0.100	0.355	0.340	6.128	0.101
		Unbalanced	G	0.111	0.115	2.979	2.970	164.850	0.119
			N	0.097	0.103	0.426	0.411	32.988	0.105
	10	Balanced	G	0.107	0.106	1.847	1.845	141.874	0.120
			N	0.074	0.076	0.336	0.329	11.417	0.077
		Unbalanced	G	0.105	0.107	1.891	1.880	162.325	0.121
			N	0.076	0.080	0.438	0.429	34.710	0.082

**Table 2:** Simulation results for the 95% coverage rate for the estimator of the 0.975 quantile. “NY”, “YY”, “YN”, “Wild” and “Naïve” are the evaluated methods described in Sect. 2.3. Column “m” represents the multiplier of the number of subjects within each study ($\:60\times\:m$ subjects each study for the balanced case, for unbalanced case $\:\left(\text{20,40,60,80,100}\right)\times\:m$ if $\:K=5$ and $\:\left(\text{20,20,40,40,60,60,80,80,100,100}\right)\times\:m$ if $\:K=10$). “Balance” represents whether the sample sizes for each study is balanced or not. “Dist” represents the distribution within each study where “G” represent Gamma distribution and “N” represent normal distribution

*m*	Study #	Balance	Dist	NY	YY	YN	Wild	Naïve
1	5	Balanced	G	0.936	0.991	0.985	1	0.948
			N	0.917	0.981	0.921	0.988	0.923
		Unbalanced	G	0.937	0.98	0.951	1	0.933
			N	0.92875	0.986	0.958	0.994	0.935
	10	Balanced	G	0.94	1	1	1	0.966
			N	0.942	0.996	0.989	1	0.948
		Unbalanced	G	0.935	0.999	0.999	1	0.96
			N	0.934	0.999	0.992	1	0.942
2	5	Balanced	G	0.933	1	1	1	0.953
			N	0.926	0.995	0.983	0.998	0.93
		Unbalanced	G	0.931	0.996	0.991	1	0.941
			N	0.945	0.995	0.989	1	0.947
	10	Balanced	G	0.943	1	1	1	0.964
			N	0.933	1	1	1	0.94
		Unbalanced	G	0.941	1	1	1	0.965
			N	0.921	1	1	1	0.931
4	5	Balanced	G	0.94	1	1	1	0.951
			N	0.923	0.997	0.992	1	0.928
		Unbalanced	G	0.956	1	1	1	0.96
			N	0.941	0.999	0.997	1	0.942
	10	Balanced	G	0.94	1	1	1	0.965
			N	0.941	1	1	1	0.941
		Unbalanced	G	0.937	1	1	1	0.967
			N	0.95	1	1	1	0.959

**Table 3:** Simulation results for the 95% coverage rate for the estimator of the 0.9 quantile. “NY”, “YY”, “YN”, “Wild” and “Naïve” are the evaluated methods described in Sect. 2.3. Column “m” represents the multiplier of the number of subjects within each study ($\:60\times\:m$ subjects each study for the balanced case, for unbalanced case $\:\left(\text{20,40,60,80,100}\right)\times\:m$ if $\:K=5$ and $\:\left(\text{20,20,40,40,60,60,80,80,100,100}\right)\times\:m$ if $\:K=10$). Column “Balance” represents whether the sample sizes for each study is balanced or not. Column “Dist” represents the distribution within each study where “G” represent Gamma distribution and “N” represent normal distribution

*m*	Study #	Balance	Dist	NY	YY	YN	Wild	Naïve
1	5	Balanced	G	0.934	1	1	1	0.991
			N	0.951	1	0.999	1	0.96
		Unbalanced	G	0.933	1	1	1	0.966
			N	0.93	1	0.999	1	0.942
	10	Balanced	G	0.922	1	1	1	1
			N	0.938	1	1	1	0.962
		Unbalanced	G	0.936	1	1	1	0.999
			N	0.931	1	1	1	0.961
2	5	Balanced	G	0.936	1	1	1	0.99
			N	0.935	1	1	1	0.956
		Unbalanced	G	0.956	1	1	1	0.972
			N	0.943	1	1	1	0.952
	10	Balanced	G	0.929	1	1	1	1
			N	0.944	1	1	1	0.964
		Unbalanced	G	0.944	1	1	1	1
			N	0.951	1	1	1	0.972
3	5	Balanced	G	0.936	1	1	1	0.985
			N	0.925	1	1	1	0.939
		Unbalanced	G	0.946	1	1	1	0.974
			N	0.958	1	1	1	0.965
	10	Balanced	G	0.937	1	1	1	1
			N	0.943	1	1	1	0.966
		Unbalanced	G	0.936	1	1	1	1
			N	0.954	1	1	1	0.978

Among all the bootstrap methods, the NY method which samples the first layer without replacement (i.e., we take all studies) and the second layer with replacement gives a mean SE closest to the empirical one and thus has the best performance. The YY method and YN method both generate estimated standard errors much larger than the empirical standard errors. This is not unexpected since the true data generating mechanism is under the fixed effects model and thus leaves the study means unchanged for each iteration. The bootstrap methods which sample the first layer of study means with replacement (YN and YY methods) tend to have larger variations since they account for additional variability in the study means compared to the fixed effects model under which the data were generated. The naïve method which resamples the pooled population with replacement has a slightly larger estimated SD compared with the NY method. We believe this is due to the fact that in the naïve method, the sample size of each individual study is changed for each bootstrap iteration, thus causing the study weights to differ across bootstrap samples and introducing extra variability. The wild bootstrap strategy tended to give extremely large standard errors under our simulation settings and was thus not optimal for the fixed effects meta-analysis.

For the coverage results of 0.975 quantile (Table 2), the NY method yields slightly smaller coverage rates (around 93%) in many simulation conditions while the naïve method has the coverage rate closer to 95% in some cases. We interpreted the slightly low coverage rate for the NY method as the effect of the extreme quantiles (0.975), since data points that are close to the tails of the distribution are likely to not be resampled. We hypothesized that the higher coverage rate (closer to 95%) for the naïve method was a result of the additional variability in the bootstrap resamples due to differing study weights counteracting the effect of the extreme quantiles (which can decrease the coverage rate). We then further conducted a simulation where we estimated the 0.9 quantile instead of the 0.975 quantile, thus significantly reducing the effect of the extreme quantiles. The results for the coverage rate can be seen in Table 3 where the coverage rate for the naïve method is very close to 100% in many cases while the NY method still has good performance. Thus, we concluded that the NY method is the optimal method for the fixed effects model. We also obtained results for the 0.025 and 0.1 quantiles from the same set of simulations used to evaluate the upper quantile. The results which are consistent with those for the upper quantile are detailed in the Supplementary materials.

In addition to comparing various bootstrap methods, we assessed the performance of the estimated 95% reference interval itself under identical simulation conditions. The results are presented in Table S6 of the Supplementary Material. For each simulation iteration, we employed the Monte Carlo method to calculate the observed coverage proportion for the estimated reference interval. We defined this as the proportion of values from the population distribution that are included in the estimated reference interval. Subsequently, the mean coverage proportion was determined by computing the mean value of the coverage proportion across the 1000 simulation replications. The theoretical length of the reference interval was derived as the difference between the 0.025 and 0.975 quantiles of the population distribution. Table S6 shows that the mean coverage rate was near 95% and the mean width of the reference interval was near the theoretical length in all scenarios.

## Case study

Chronic liver disease is a condition associated with important morbidity and mortality, often from progression to liver fibrosis (scarring) which eventually leads to cirrhosis, an end-stage complication with poor prognosis. It is challenging to monitor patients for progression of this fibrosis because of paucity of symptoms [30, 31]. The gold standard for assessing liver fibrosis is a liver biopsy, which usually takes a 1–3 cm specimen from the liver for examination [32]. This invasive procedure is burdensome to patients and can have serious complications, including death in some rare situations [33]. The FibroScan^®^ is newly developed noninvasive approach for assessing liver stiffness (a surrogate of fibrosis) with greater safety and convenience. Liver stiffness measurements of 8 and 12.5 kPa using the FibroScan^®^ represent generally accepted cut-off values for F3 fibrosis (advanced fibrosis) and F4 fibrosis (cirrhosis) [31]. However, there is no consensus on the reference interval for liver stiffness in children.

Li et al., [34] recently performed a meta-analysis with individual participant data to estimate a reference interval for the liver stiffness of healthy children. Specifically, they included studies with apparently healthy individuals (no obesity or known liver disease) and age less than 18 years old. Additionally, only studies that used a standard small (S1 and S2) or standard medium (M) probe were included. With these criteria, they identified 5 studies with a total sample size 652, in which 588 (90.2%) were in the older group (larger than 3 years old) and 64 (9.8%) were in the younger group (smaller than 3 years old). They defined the reference interval (which is 87.5% instead of 95%) as bounded by 2.5th and 90th percentiles of the liver stiffness measure which corresponded to 2.45–5.56 kPa for healthy children in their dataset. Here, we re-analyze data from the older group of their primary analysis cohort (age ≥ 3 years; 588 individuals from 5 studies) using the proposed quantile regression method to estimate the reference interval for the liver stiffness measure both overall as well as for specific values of covariates. To be consistent with their analysis results, we use the 0.025 and 0.9 quantile as the lower and upper bound of the reference interval. This will lead to an 87.5% reference interval instead of the conventional 95% reference interval.

**Fig. 1:**
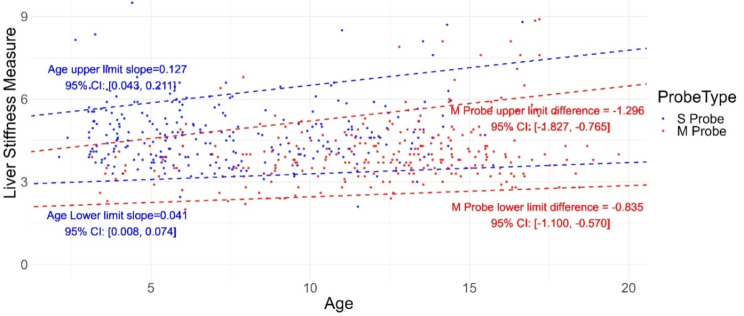
Scatter plot for relationship of the liver stiffness measure and BMI. Two dashed lines at the top are the fitted 97.5% quantile regression lines, two dashed lines at the bottom are the fitted 2.5% quantile regression lines. Red dashed lines are the reference interval for M probe and the blue dashed lines are the reference interval for the S probe

We focused on the following covariates identified in the original study: race (Caucasian or other), sex (male and female), probe type (S probe or M probe), BMI, age, and sedation status (whether the child was sedated during the measurement). Starting with the full model containing all six prespecified covariates, we employed backward selection based on the p-value of each covariate (covariates with the largest p-value will be excluded unless it is smaller than 0.05) to obtain the final multivariable quantile regression models for the upper and lower bounds of the reference interval. Despite selecting the upper and lower bounds separately, the final models both included age and probe type as the selected covariates (presented in Table 4). Figure 1 shows the estimated upper and lower limits of the reference interval by age and probe type. The results indicate that a one-year difference in age was associated with a 0.127 kPa higher upper limit of the reference interval and a 0.041 kPa higher lower limit of the reference interval, suggesting that older patients are expected to have higher liver stiffness measurements, while still being considered healthy. Similarly, the M probe type was associated with upper and lower reference interval limits of 1.296 kPa and 0.835 kPa less than the S probe type, respectively, indicating that healthy patients using the S probe are likely to have higher liver stiffness measures.

**Table 4:** Multivariable quantile regression results for reference interval limits. Lower and upper bound are estimated as 0.025 and 0.9 quantile from the regression. Standard errors are estimated using the NY bootstrap strategy

Bound	Covariates	Coefficients	Standard Error	*P*-value
Lower	**M probe**	-0.834	0.135	< 0.001
	**Age**	0.041	0.017	0.017
Upper	**M probe**	-1.296	0.271	< 0.001
	**Age**	0.127	0.043	0.003
Median	**M probe**	-0.807	0.119	< 0.001
	**Age**	0.030	0.015	0.046

The observed impact of age appears to be more pronounced on the upper limit of the reference interval. To investigate this further, we explored its effect on the median liver stiffness measurement (0.5 quantile); the findings presented in Table 4. While age remains significantly associated with median liver stiffness, its slope (0.03) is considerably smaller than for the upper reference limit (0.127). This suggests that the increase in age predominantly affects the upper bounds of the reference interval rather than the median of the population.

## Discussion

In this paper, we introduce quantile regression as a method for estimating the reference interval from an IPD meta-analysis without assuming a specific within-study distribution. We adopted the fixed effects model which assumes the samples from different studies are independently distributed and they together form a representative population. For the fixed effects model, we found that the best performing bootstrap method keep the studies fixed and only randomly sampling subjects with replacement within each study to incorporate the clustered data structure. This is different from previous recommendations under the random effects model, where the optimal choice is to sample the first layer with replacement and the second layer without replacement (the YN method; see Ren et al., 2010^28^) As illustrated in Table 2, other bootstrap methods can lead to likely overestimated standard errors, greatly reducing power. If the meta-analysis has a large number of studies (usually larger than 15), a random effects model can be applied to estimate the between study heterogeneity [35]. Then, the bootstrap method should be modified to reflect random effects modeling assumption that the study means are sampled from some distribution. Further study can explore the best bootstrap method for the random effects model in the meta-analysis setting where the cluster number is relatively small.

Compared with the methods for estimating the reference interval from aggregate data meta-analysis, using the quantile regression with IPD not only has flexible distributional assumptions but also enables researchers to explore the heterogeneity of the reference interval within the population. In the case study, we identified several covariates (such as the type of used probe) that may potentially influence the upper or lower bound of the reference interval for the liver stiffness measure. These findings allow for the creation of personalized reference intervals based on individual patient characteristics, increasing the accuracy of estimated reference intervals in medical practice. For simplicity, we used backwards selection based on the p-value to identify covariates to include in the final case study models. In practice, it may be advisable to consider other methods of variable selection, such as those using the AIC. Further work could also explore the use of penalized quantile regression methods [36–38]. Although the estimation of the subject specific reference interval depends on the correct specification of the quantile regression model, researchers have shown the robustness of quantile regression in estimating the conditional quantile [39].

One limitation of this method for estimating the reference interval is that it is highly dependent on the included population. With the use of the fixed effects meta-analysis model, we implicitly assume that the population from the included studies reflects the true population of interest. Thus, the estimated reference interval is based on the aggregated study populations which, in some scenarios, may not reflect the exact population of interest. Although this may be mitigated by including covariates, researchers should carefully consider the target population.

## Electronic supplementary material

Below is the link to the electronic supplementary material.

Supplementary Material 1

## Data Availability

The datasets used and/or analyzed during the current study are available from the corresponding author on reasonable request.
