# Supplementary material for: Estimating reference intervals from an IPD meta-analysis using quantile regression
Source: BMC Med Res Methodol. 2024 Oct 26;24:251. doi: 10.1186/s12874-024-02378-0 (PMC11514908; doi:10.1186/s12874-024-02378-0)
Supplement: Supplementary file 1 — Supplementary Material 1 [file 12874_2024_2378_MOESM1_ESM.docx]

**Supplementary materials for estimating subject-specific reference intervals from a meta-analysis using quantile regression**

**S.1 Derivation of Quantile Regression**

Consider a random variable $Y$ with the cumulative distribution function:

$$F\left( y \right)=Pr \left( Y\leq y \right).$$

We can define its $\tau$-th quantile for any $\tau\in(0,1)$:

$$Q\left( \tau\right)=\inf\left\{ y:F\left( y \right)\geq\tau\right\} .$$

The quantiles $Q(\tau)$ can be written as the following optimization problem [8]:

$$\hat{Q_{\tau}}=\arg E[\rho_{\tau}(Y-\xi)]$$

Where $\rho_{\tau}\left( u \right)=u(\tau-I(u<0))$ is the “check function” with

$$\rho_{\tau}\left( u \right)=\left\{ \begin{aligned} \tau u, u\geq0 \\ (\tau-1)u, &u<0 \end{aligned} \right.$$

Then, the sample quantile can be calculated using the sample data $\left\{ y_{1},\ldots{,y}_{n} \right\}\sim^{i.i.d.}Y$ as:

$$\hat{Q_{\tau}}=\arg\sum_{i=1}^{n} \rho_{\tau}(y_{i}-\xi)$$

Assume the covariates for each individual $i$ are $\boldsymbol{x}_{\boldsymbol{i}}$, $Q_{Y}\left( \tau|\boldsymbol{x}_{\boldsymbol{i}},\beta_{\tau} \right)=\boldsymbol{x}_{\boldsymbol{i}}^{T}\boldsymbol{\beta}_{\tau}$ is the $\tau$-th conditional quantile of $Y$given the predictor $\boldsymbol{X}=\boldsymbol{x}_{\boldsymbol{i}}$, then the parameter $\boldsymbol{\beta}$ can be estimated by minimizing the following quantity:

$$\hat{\boldsymbol{\beta}}=\arg\sum_{i=1}^{n} \rho_{\tau}(y_{i}-\boldsymbol{x}_{\boldsymbol{i}}^{T}\boldsymbol{\beta}).$$

**S.02 Additional Simulation Results**

**Table S1**: Simulation results for the estimated standard error for the estimator of the 0.9 quantile. The “Emp” SE is the standard deviation of the simulated quantiles. Column “m” represents the multiplier of the number of subjects within each study. “Balance” represents whether the sample sizes for each study is balanced or not. C “Dist” represents the distribution within each study where “G” represent Gamma distribution and “N” represent Normal distribution.

| $\boldsymbol{m}$ | Study Number | Balance | Dist | Emp | NY | YY | YN | Wild | Naïve |
| --- | --- | --- | --- | --- | --- | --- | --- | --- | --- |
| 1 | 5 | Balanced | G | 0.159 | 0.166 | 2.680 | 2.672 | 23.393 | 0.229 |
|  |  |  | N | 0.106 | 0.112 | 0.425 | 0.410 | 0.576 | 0.118 |
|  |  | Unbalanced | G | 0.143 | 0.136 | 3.007 | 3.005 | 34.013 | 0.155 |
|  |  |  | N | 0.116 | 0.118 | 0.559 | 0.547 | 6.241 | 0.125 |
|  | 10 | Balanced | G | 0.155 | 0.158 | 1.889 | 1.871 | 23.330 | 0.347 |
|  |  |  | N | 0.089 | 0.089 | 0.472 | 0.463 | 2.510 | 0.099 |
|  |  | Unbalanced | G | 0.147 | 0.150 | 1.914 | 1.907 | 28.029 | 0.305 |
|  |  |  | N | 0.095 | 0.097 | 0.630 | 0.624 | 7.477 | 0.114 |
| 2 | 5 | Balanced | G | 0.118 | 0.117 | 2.674 | 2.671 | 23.871 | 0.158 |
|  |  |  | N | 0.079 | 0.080 | 0.412 | 0.405 | 0.465 | 0.083 |
|  |  | Unbalanced | G | 0.093 | 0.097 | 3.007 | 3.005 | 34.541 | 0.110 |
|  |  |  | N | 0.082 | 0.082 | 0.543 | 0.535 | 6.907 | 0.087 |
|  | 10 | Balanced | G | 0.118 | 0.112 | 1.861 | 1.853 | 23.088 | 0.247 |
|  |  |  | N | 0.061 | 0.063 | 0.463 | 0.461 | 2.410 | 0.069 |
|  |  | Unbalanced | G | 0.101 | 0.103 | 1.903 | 1.899 | 28.089 | 0.214 |
|  |  |  | N | 0.067 | 0.068 | 0.622 | 0.620 | 7.766 | 0.080 |
| 3 | 5 | Balanced | G | 0.093 | 0.095 | 2.672 | 2.673 | 24.041 | 0.127 |
|  |  |  | N | 0.067 | 0.065 | 0.408 | 0.402 | 0.329 | 0.068 |
|  |  | Unbalanced | G | 0.075 | 0.079 | 3.012 | 3.005 | 34.921 | 0.090 |
|  |  |  | N | 0.065 | 0.067 | 0.539 | 0.533 | 6.896 | 0.071 |
|  | 10 | Balanced | G | 0.092 | 0.091 | 1.848 | 1.841 | 23.280 | 0.202 |
|  |  |  | N | 0.050 | 0.051 | 0.461 | 0.460 | 2.420 | 0.056 |
|  |  | Unbalanced | G | 0.085 | 0.086 | 1.902 | 1.894 | 28.166 | 0.176 |
|  |  |  | N | 0.054 | 0.056 | 0.619 | 0.616 | 7.717 | 0.065 |

**Table S2**: Simulation results for the estimated standard error for the estimator of the 0.025 quantile. The “Emp” SE is the standard deviation of the simulated quantiles. Column “m” represents the multiplier of the number of subjects within each study. “Balance” represents whether the sample sizes for each study is balanced or not. “Dist” represents the distribution within each study where “G” represent Gamma distribution and “N” represent Normal distribution.

| $\boldsymbol{m}$ | Study Number | Balance | Dist | Emp | NY | YY | YN | Wild | Naïve |
| --- | --- | --- | --- | --- | --- | --- | --- | --- | --- |
| 1 | 5 | Balance | G | 0.161 | 0.168 | 0.621 | 0.601 | 3.099 | 0.173 |
|  |  |  | N | 0.196 | 0.203 | 0.833 | 0.812 | 0.538 | 0.212 |
|  |  | Unbalance | G | 0.155 | 0.162 | 0.701 | 0.681 | 5.419 | 0.165 |
|  |  |  | N | 0.182 | 0.189 | 0.861 | 0.838 | 0.536 | 0.194 |
|  | 10 | Balance | G | 0.121 | 0.126 | 0.440 | 0.424 | 2.386 | 0.133 |
|  |  |  | N | 0.173 | 0.180 | 1.501 | 1.494 | 0.769 | 0.207 |
|  |  | Unbalance | G | 0.118 | 0.121 | 0.507 | 0.493 | 2.806 | 0.125 |
|  |  |  | N | 0.169 | 0.177 | 1.420 | 1.414 | 0.777 | 0.204 |
| 2 | 5 | Balance | G | 0.113 | 0.119 | 0.605 | 0.592 | 2.854 | 0.122 |
|  |  |  | N | 0.141 | 0.140 | 0.810 | 0.800 | 0.545 | 0.146 |
|  |  | Unbalance | G | 0.111 | 0.114 | 0.685 | 0.674 | 5.299 | 0.116 |
|  |  |  | N | 0.129 | 0.134 | 0.850 | 0.840 | 0.588 | 0.138 |
|  | 10 | Balance | G | 0.090 | 0.089 | 0.429 | 0.419 | 2.504 | 0.094 |
|  |  |  | N | 0.123 | 0.127 | 1.494 | 1.486 | 0.764 | 0.146 |
|  |  | Unbalance | G | 0.083 | 0.085 | 0.493 | 0.487 | 2.676 | 0.088 |
|  |  |  | N | 0.119 | 0.126 | 1.411 | 1.412 | 0.802 | 0.144 |
| 3 | 5 | Balance | G | 0.093 | 0.095 | 0.596 | 0.589 | 2.684 | 0.097 |
|  |  |  | N | 0.114 | 0.114 | 0.807 | 0.799 | 0.552 | 0.119 |
|  |  | Unbalance | G | 0.086 | 0.091 | 0.677 | 0.671 | 5.188 | 0.093 |
|  |  |  | N | 0.110 | 0.111 | 0.848 | 0.841 | 0.608 | 0.115 |
|  | 10 | Balance | G | 0.071 | 0.072 | 0.422 | 0.414 | 2.574 | 0.076 |
|  |  |  | N | 0.100 | 0.103 | 1.493 | 1.489 | 0.781 | 0.117 |
|  |  | Unbalance | G | 0.068 | 0.068 | 0.491 | 0.484 | 2.585 | 0.071 |
|  |  |  | N | 0.101 | 0.103 | 1.416 | 1.413 | 0.798 | 0.117 |

**Table S3**: Simulation results for the 95% coverage rate for the estimator of the 0.025 quantile. Column “m” represents the multiplier of the number of subjects within each study. “Balance” represents whether the sample sizes for each study is balanced or not. “Dist” represents the distribution within each study where “G” represent Gamma distribution and “N” represent Normal distribution.

| $\boldsymbol{m}$ | Study Number | Balance | Dist | NY | YY | YN | Wild | Naïve |
| --- | --- | --- | --- | --- | --- | --- | --- | --- |
| 1 | 5 | Balance | G | 0.935 | 0.992 | 0.96 | 0.998 | 0.938 |
|  |  |  | N | 0.922 | 0.995 | 0.974 | 0.999 | 0.93 |
|  |  | Unbalance | G | 0.924 | 0.989 | 0.962 | 0.999 | 0.93 |
|  |  |  | N | 0.935 | 0.98625 | 0.97875 | 1 | 0.94 |
|  | 10 | Balance | G | 0.941 | 0.995 | 0.994 | 1 | 0.95 |
|  |  |  | N | 0.939 | 1 | 1 | 1 | 0.973 |
|  |  | Unbalance | G | 0.94 | 0.995 | 0.992 | 1 | 0.943 |
|  |  |  | N | 0.939 | 0.999 | 0.998 | 1 | 0.965 |
| 2 | 5 | Balance | G | 0.946 | 1 | 0.996 | 1 | 0.954 |
|  |  |  | N | 0.926 | 1 | 1 | 1 | 0.931 |
|  |  | Unbalance | G | 0.938 | 1 | 0.994 | 1 | 0.943 |
|  |  |  | N | 0.942 | 0.999 | 0.999 | 1 | 0.948 |
|  | 10 | Balance | G | 0.941 | 1 | 1 | 1 | 0.95 |
|  |  |  | N | 0.949 | 1 | 1 | 1 | 0.974 |
|  |  | Unbalance | G | 0.941 | 1 | 1 | 1 | 0.949 |
|  |  |  | N | 0.944 | 1 | 1 | 1 | 0.97 |
| 3 | 5 | Balance | G | 0.944 | 1 | 1 | 1 | 0.95 |
|  |  |  | N | 0.937 | 1 | 1 | 1 | 0.945 |
|  |  | Unbalance | G | 0.947 | 0.999 | 0.999 | 1 | 0.954 |
|  |  |  | N | 0.942 | 1 | 1 | 1 | 0.958 |
|  | 10 | Balance | G | 0.944 | 1 | 1 | 1 | 0.955 |
|  |  |  | N | 0.941 | 1 | 1 | 1 | 0.962 |
|  |  | Unbalance | G | 0.947 | 1 | 1 | 1 | 0.956 |
|  |  |  | N | 0.944 | 1 | 1 | 1 | 0.969 |

**Table S4**: Simulation results for the estimated standard error for the estimator of the 0.1 quantile. The “Emp” SE is the standard deviation of the simulated quantiles. Column “m” represents the multiplier of the number of subjects within each study. “Balance” represents whether the sample sizes for each study is balanced or not. “Dist” represents the distribution within each study where “G” represent Gamma distribution and “N” represent Normal distribution.

| $\boldsymbol{m}$ | Study Number | Balance | Dist | Emp | NY | YY | YN | Wild | Naïve |
| --- | --- | --- | --- | --- | --- | --- | --- | --- | --- |
| 1 | 5 | Balance | G | 0.122 | 0.119 | 0.640 | 0.631 | 2.468 | 0.132 |
|  |  |  | N | 0.128 | 0.133 | 0.800 | 0.787 | 0.738 | 0.156 |
|  |  | Unbalance | G | 0.115 | 0.116 | 0.838 | 0.830 | 4.988 | 0.125 |
|  |  |  | N | 0.123 | 0.127 | 0.850 | 0.837 | 0.809 | 0.144 |
|  | 10 | Balance | G | 0.089 | 0.089 | 0.509 | 0.502 | 1.697 | 0.105 |
|  |  |  | N | 0.163 | 0.157 | 1.358 | 1.347 | 1.676 | 0.354 |
|  |  | Unbalance | G | 0.087 | 0.086 | 0.574 | 0.567 | 2.251 | 0.098 |
|  |  |  | N | 0.144 | 0.141 | 1.271 | 1.265 | 1.492 | 0.287 |
| 2 | 5 | Balance | G | 0.080 | 0.084 | 0.636 | 0.630 | 2.503 | 0.093 |
|  |  |  | N | 0.095 | 0.094 | 0.791 | 0.785 | 0.736 | 0.110 |
|  |  | Unbalance | G | 0.080 | 0.081 | 0.828 | 0.823 | 5.039 | 0.088 |
|  |  |  | N | 0.090 | 0.090 | 0.845 | 0.839 | 0.853 | 0.101 |
|  | 10 | Balance | G | 0.062 | 0.063 | 0.504 | 0.499 | 1.578 | 0.074 |
|  |  |  | N | 0.113 | 0.114 | 1.345 | 1.338 | 1.678 | 0.255 |
|  |  | Unbalance | G | 0.060 | 0.060 | 0.567 | 0.565 | 2.171 | 0.069 |
|  |  |  | N | 0.096 | 0.100 | 1.264 | 1.265 | 1.511 | 0.206 |
| 3 | 5 | Balance | G | 0.066 | 0.068 | 0.630 | 0.627 | 2.464 | 0.075 |
|  |  |  | N | 0.078 | 0.077 | 0.789 | 0.785 | 0.745 | 0.089 |
|  |  | Unbalance | G | 0.067 | 0.065 | 0.825 | 0.825 | 5.056 | 0.070 |
|  |  |  | N | 0.071 | 0.073 | 0.842 | 0.839 | 0.872 | 0.082 |
|  | 10 | Balance | G | 0.050 | 0.051 | 0.499 | 0.496 | 1.565 | 0.060 |
|  |  |  | N | 0.089 | 0.092 | 1.345 | 1.340 | 1.683 | 0.210 |
|  |  | Unbalance | G | 0.049 | 0.049 | 0.567 | 0.563 | 2.112 | 0.056 |
|  |  |  | N | 0.080 | 0.083 | 1.266 | 1.264 | 1.516 | 0.169 |

**Table S5**: Simulation results for the 95% coverage rate for the estimator of the 0.1 quantile. Column “m” represents the multiplier of the number of subjects within each study. “Balance” represents whether the sample sizes for each study is balanced or not. “Dist” represents the distribution within each study where “G” represent Gamma distribution and “N” represent Normal distribution.

| $\boldsymbol{m}$ | Study Number | Balance | Dist | NY | YY | YN | Wild | Naïve |
| --- | --- | --- | --- | --- | --- | --- | --- | --- |
| 1 | 5 | Balance | G | 0.938 | 1 | 1 | 1 | 0.96 |
|  |  |  | N | 0.934 | 1 | 1 | 1 | 0.971 |
|  |  | Unbalance | G | 0.934 | 1 | 1 | 1 | 0.957 |
|  |  |  | N | 0.941 | 1 | 1 | 1 | 0.968 |
|  | 10 | Balance | G | 0.938 | 1 | 1 | 1 | 0.976 |
|  |  |  | N | 0.912 | 1 | 1 | 1 | 1 |
|  |  | Unbalance | G | 0.941 | 1 | 1 | 1 | 0.967 |
|  |  |  | N | 0.935 | 1 | 1 | 1 | 0.999 |
| 2 | 5 | Balance | G | 0.947 | 1 | 1 | 1 | 0.969 |
|  |  |  | N | 0.933 | 1 | 1 | 1 | 0.965 |
|  |  | Unbalance | G | 0.952 | 1 | 1 | 1 | 0.964 |
|  |  |  | N | 0.927 | 1 | 1 | 1 | 0.959 |
|  | 10 | Balance | G | 0.951 | 1 | 1 | 1 | 0.977 |
|  |  |  | N | 0.94 | 1 | 1 | 1 | 1 |
|  |  | Unbalance | G | 0.943 | 1 | 1 | 1 | 0.968 |
|  |  |  | N | 0.956 | 1 | 1 | 1 | 1 |
| 3 | 5 | Balance | G | 0.953 | 1 | 1 | 1 | 0.97 |
|  |  |  | N | 0.929 | 1 | 1 | 1 | 0.967 |
|  |  | Unbalance | G | 0.928 | 1 | 1 | 1 | 0.944 |
|  |  |  | N | 0.947 | 1 | 1 | 1 | 0.972 |
|  | 10 | Balance | G | 0.951 | 1 | 1 | 1 | 0.973 |
|  |  |  | N | 0.945 | 1 | 1 | 1 | 1 |
|  |  | Unbalance | G | 0.929 | 1 | 1 | 1 | 0.968 |
|  |  |  | N | 0.954 | 1 | 1 | 1 | 1 |

**Table S6**: Simulation results for the estimated reference interval under different simulation conditions. Column “m” represents the multiplier of the number of subjects within each study. “Balance” represents whether the sample sizes for each study are balanced or not. “Dist” represents the distribution within each study where “G” represents Gamma distribution and “N” represent Normal distribution. We display the mean coverage proportion for the estimated 95% reference interval, the length of the estimated reference interval and the theoretical length calculated under Monte Carlo method.

| $\boldsymbol{m}$ | Study Number | Balance | Dist | Mean  Coverage | RI Length | Theoretical Length |
| --- | --- | --- | --- | --- | --- | --- |
| 1 | 5 | Balance | G | 0.948 | 10.875 | 10.882 |
|  |  |  | N | 0.947 | 6.225 | 6.221 |
|  |  | Unbalance | G | 0.946 | 11.203 | 11.230 |
|  |  |  | N | 0.947 | 6.292 | 6.296 |
|  | 10 | Balance | G | 0.948 | 10.990 | 11.016 |
|  |  |  | N | 0.947 | 9.530 | 9.586 |
|  |  | Unbalance | G | 0.949 | 11.206 | 11.216 |
|  |  |  | N | 0.947 | 9.350 | 9.409 |
| 2 | 5 | Balance | G | 0.949 | 10.877 | 10.881 |
|  |  |  | N | 0.946 | 6.192 | 6.243 |
|  |  | Unbalance | G | 0.948 | 11.218 | 11.228 |
|  |  |  | N | 0.946 | 6.257 | 6.305 |
|  | 10 | Balance | G | 0.949 | 11.006 | 11.013 |
|  |  |  | N | 0.948 | 9.554 | 9.585 |
|  |  | Unbalance | G | 0.949 | 11.208 | 11.215 |
|  |  |  | N | 0.948 | 9.376 | 9.406 |
| 3 | 5 | Balance | G | 0.949 | 10.874 | 10.886 |
|  |  |  | N | 0.949 | 6.229 | 6.239 |
|  |  | Unbalance | G | 0.949 | 11.224 | 11.228 |
|  |  |  | N | 0.949 | 6.294 | 6.307 |
|  | 10 | Balance | G | 0.949 | 11.011 | 11.019 |
|  |  |  | N | 0.949 | 9.564 | 9.581 |
|  |  | Unbalance | G | 0.950 | 11.212 | 11.217 |
|  |  |  | N | 0.949 | 9.391 | 9.404 |
